# Efficacy and acceptability of lurasidone for bipolar depression: a systematic review and dose–response meta-analysis

**DOI:** 10.1136/bmjment-2024-301165

**Published:** 2024-11-18

**Authors:** Yu-Wei Lin, Yang-Chieh Brian Chen, Kuo-Chuan Hung, Chih-Sung Liang, Ping-Tao Tseng, Andre F Carvalho, Eduard Vieta, Marco Solmi, Edward Chia-Cheng Lai, Pao-Yen Lin, Chih-Wei Hsu, Yu-Kang Tu

**Affiliations:** 1 Department of Psychiatry, Kaohsiung Chang Gung Memorial Hospital and Chang Gung University College of Medicine, Kaohsiung, Taiwan; 2 Department of Anesthesiology, Chi Mei Medical Center, Tainan, Taiwan; 3 Department of Psychiatry, Beitou Branch, Tri-Service General Hospital, National Defense Medical Center, Taipei, Taiwan; 4 Department of Psychiatry, National Defense Medical Center, Taipei, Taiwan; 5 Prospect Clinic for Otorhinolaryngology & Neurology, Kaohsiung, Taiwan; 6 Institute of Biomedical Sciences, National Sun Yat-sen University, Kaohsiung, Taiwan; 7 Department of Psychology, College of Medical and Health Science, Asia University, Taichung, Taiwan; 8 Institute of Precision Medicine, National Sun Yat-sen University, Kaohsiung City, Taiwan; 9 Innovation in Mental and Physical Health and Clinical Treatment (IMPACT) Strategic Research Centre, School of Medicine, Barwon Health, Deakin University, Geelong, Victoria, Australia; 10 Bipolar and Depressive Disorders Unit, Hospital Clinic, IDIBAPS, CIBERSAM, Institute of Neuroscience, University of Barcelona, Barcelona, Catalonia, Spain; 11 Department of Psychiatry, University of Ottawa, Ottawa, Ontario, Canada; 12 Department of Mental Health, The Ottawa Hospital, Ottawa, Ontario, Canada; 13 Ottawa Hospital Research Institute (OHRI), Clinical Epidemiology Program, University of Ottawa, Ottawa, Ontario, Canada; 14 School of Epidemiology and Public Health, Faculty of Medicine, University of Ottawa, Ottawa, Ontario, Canada; 15 Department of Child and Adolescent Psychiatry, Charité Universitätsmedizin, Berlin, Germany; 16 School of Pharmacy, Institute of Clinical Pharmacy and Pharmaceutical Sciences, College of Medicine, National Cheng Kung University, Tainan, Taiwan; 17 Population Health Data Center, National Cheng Kung University, Tainan, Taiwan; 18 Institute of Health Data Analytics & Statistics, College of Public Health, National Taiwan University, Taipei, Taiwan; 19 Department of Dentistry, National Taiwan University Hospital, Taipei, Taiwan

**Keywords:** Depression, PSYCHIATRY, Depression & mood disorders

## Abstract

**Question:**

The optimal dose of lurasidone for bipolar depression is unclear. This study examined its dose–response relationship for efficacy, acceptability, and metabolic/endocrine profiles.

**Study selection and analysis:**

Five databases and grey literature published until 1 August 2024, were systematically reviewed. The outcomes included efficacy (changes in depression, anxiety, clinical global impression, disability and quality of life), acceptability (dropout, manic switch, suicidality and side effects) and metabolic/endocrine profiles (changes in body weight, glucose, lipid and prolactin levels). Effect sizes were calculated using a one-step dose–response meta-analysis, expressed as standardised mean differences (SMDs), risk ratios (RRs) and mean differences (MDs) with 95% CIs.

**Findings:**

Five randomised clinical trials (2032 patients, mean treatment duration 6 weeks) indicated that the optimal therapeutic dose of lurasidone (40–60 mg) improved depression (50 mg: SMD −0.60 (95% CI −0.30, –0.89)), anxiety (50 mg: −0.32 (95% CI −0.21, –0.42)), clinical global impression (50 mg: −0.67 (95% CI −0.30, –1.03)) and disability (50 mg: −0.38 (95% CI −0.08, –0.69)). Side effects increased with higher doses (50 mg: RR 1.15 (95% CI 1.05, 1.25); 100 mg: 1.18 (95% CI 1.02, 1.36)), but dropout, manic switch and suicidality did not show a dose–effect relationship. Weight increased at doses<60 mg (40 mg: MD 0.38 (95% CI 0.16, 0.60) kg), while blood glucose levels rose at doses>70 mg (100 mg: 3.16 (95% CI 0.76, 5.57) mg/dL). Prolactin levels increased in both males (50 mg: 3.21 (95% CI 1.59, 4.84) ng/mL; 100 mg: 5.61 (95% CI 2.42, 8.81)) and females (50 mg: 6.64 (95% CI 3.50, 9.78); 100 mg: 5.33 (95% CI 0.67, 10.00)).

**Conclusions:**

A daily dose of 40–60 mg of lurasidone is a reasonable choice for bipolar depression treatment.

**Trial registration number:**

INPLASY202430069.

**WHAT IS ALREADY KNOWN ON THIS TOPIC**

- In clinical guidelines, lurasidone is recommended as a first-line drug for bipolar depression. We searched the PubMed, EMBASE, Cochrane CENTRAL, ScienceDirect and ClinicalTrials.gov databases from their respective inception dates to 1 August 2024. However, no consensus regarding the optimal dosing recommendations of lurasidone for the treatment of bipolar depression was available.

**WHAT THIS STUDY ADDS**

- Our dose–response meta-analysis suggested that a daily dose of lurasidone within the range of 40–60 mg achieves optimal outcomes in alleviating depression, improving anxiety and reducing disability. Although dose escalation was associated with an increased incidence of side effects, no significant dose-dependent associations were observed for the main outcomes, such as dropout rates, switch from depression to mania or occurrence of suicidal events.

**HOW THIS STUDY MIGHT AFFECT RESEARCH, PRACTICE OR POLICY**

- For patients with bipolar depression, a daily dose of lurasidone in the range of 40–60 mg is recommended, although adjustments can be made based on individual patient considerations.

## Background

 Bipolar disorder has a reported lifetime prevalence of approximately 2%,^1^ and is diagnosed when patients experience manic or hypomanic episodes.^2^ While the disease course does not always involve major depressive episodes,^3^ at least 70% of patients with bipolar disorder do experience depressive symptoms,^4^ such as decreased interest, lack of energy, feelings of guilt and suicidal ideation. This condition often severely affects the patient’s life, resulting in impaired social functioning.^5^ Therefore, treatment of the depressive phase of bipolar disorder is crucial.

Lithium, quetiapine and lurasidone are the currently recommended first-line treatments for bipolar depression according to several medical guidelines, including the Canadian Network for Mood and Anxiety Treatments and the International Society for Bipolar Disorders,^6^ the International College of Neuro-Psychopharmacology^7^ and the Taiwanese Society of Biological Psychiatry and Neuropsychopharmacology.^8^ Most of these guidelines also provide recommended dosages for these medications. For example, lithium should be maintained at therapeutic serum concentrations of at least 0.8 mEq/L,^6 8^ and quetiapine should be administered at a dose of at least 300 mg.^6^,^8^ However, there is no consensus on the dosage of lurasidone used to treat bipolar depression. Current recommendations either lack specific dosing guidance^6^ or only provide a broad range of doses within safety thresholds (20–120 mg).^7 8^ This underscores the current lack of clarity regarding the optimal use of lurasidone. Given this ambiguity and the potentially more restrictive dosage range for treating bipolar depression, a thorough analysis of dosing patterns in this area is warranted.

Dose–response meta-analysis is an analytical method to address this issue, and several previous studies have adopted this method to analyse psychotropic drugs, such as antipsychotics for schizophrenia,^9^ antidepressants for acute depression^10^ and zuranolone for postpartum depression.^11^ This analytical approach studies the relationship between drug dose and therapeutic effect, providing clinicians with important insights into the optimal dose range to achieve desired drug outcomes.

## Objective

To address the gap in knowledge regarding the use of lurasidone in the treatment of bipolar depression, this study aimed to include randomised controlled trials (RCTs) in a systematic review using a dose–response meta-analysis approach. Our investigation focused on assessing the efficacy of lurasidone in addressing depression and related concerns, while also evaluating the acceptability of lurasidone treatment through the analysis of dropouts, side effects and laboratory test results.

### Study selection and analysis

#### Search strategy and study selection

This study adhered to the 2020 guidelines for systematic reviews and meta-analyses (Preferred Reporting Items for Systematic review and Meta-Analysis (PRISMA) 2020, online supplemental eTable 1).^12^ The protocol was registered with the International Platform of Registered Systematic Review and Meta-Analysis Protocols.

A comprehensive literature search was conducted in PubMed, EMBASE, Cochrane CENTRAL, ScienceDirect, ClinicalTrials.gov and the grey literature, from the respective database inception dates up to 1 August 2024. The search employed keywords such as (‘lurasidone’ OR ‘SM-13496’) AND (‘depress*’ OR ‘bipolar’ OR ‘affective’ OR ‘mood’) without any restrictions on language or geographical region. Additionally, the bibliography and reference lists of relevant articles were reviewed to identify further relevant studies. The specific search strings are provided in online supplemental eTable 2. The Population, Intervention, Comparison, Outcomes and Study framework was followed to include articles: (1) patients were diagnosed with a major depressive episode of bipolar disorder; (2) patients were treated with lurasidone; (3) comparison with placebo was required; (4) outcomes included changes in depression severity, anxiety severity, overall severity, disability, quality of life, dropout rates, side effect rates, metabolic changes, and endocrine changes after lurasidone treatment; and (5) study design was an RCT.

Our study aimed to identify RCTs that investigated the efficacy and acceptability of lurasidone for treating bipolar depression. The inclusion criteria were as follows: (1) Only RCTs comparing lurasidone with placebo or different doses of lurasidone (at least two arms, with or without co-administration) were eligible. In this study, placebo was defined as zero dose of lurasidone. (2) Participants were required to have a diagnosis of bipolar depression based on established criteria (eg, Diagnostic and Statistical Manual of Mental Disorders). (3) RCTs were required to quantify the severity of depression, using a validated scale, before and after lurasidone administration (eg, the Montgomery-Asberg Depression Rating Scale (MADRS)). The following studies were excluded: (1) Studies comparing lurasidone to other active treatments without a placebo control group, because these could not provide data on the equivalent dose of lurasidone. (2) Studies with participants with a diagnosis other than bipolar disorder, such as schizophrenia. (3) Studies that did not report outcomes related to depressive symptoms. (4) Duplicated data from a research protocol. If multiple publications had the same research origin, only the most comprehensive report with the largest sample size was included.

Two independent reviewers (Y-WL and C-WH) initially screened titles and abstracts to identify potentially relevant studies. Both the reviewers independently conducted full-text reviews of the selected articles (inter-rater reliability=0.83). A third reviewer (P-TT) resolved any disagreements that arose during the full-text review.

### Data extraction and quality assessment

Two independent reviewers (Y-WL and C-WH) extracted the data from each eligible study (inter-rater reliability=0.93). These data included publication information, study design, participant characteristics (diagnostic criteria, age, sex and number of cases), treatment protocols (dosing regimen and treatment duration), symptom assessment tools and regions. Our analysis focused on efficacy and acceptability. For the efficacy assessment, the primary outcome measure of efficacy was the change in depression severity. The MADRS was selected as the primary measure, and if the study did not employ the MADRS, an alternative depression scale was considered, such as the Hamilton Depression Rating Scale. Secondary efficacy outcomes included: (1) anxiety severity, such as a change in the Hamilton Anxiety Rating Scale scores; (2) overall illness severity, such as a change in clinical global impression (CGI) scores; (3) disability, such as a change in the Sheehan Disability Scale scores; and (4) quality of life (QOL). The primary outcome measure for the acceptability assessment was the dropout rate. The participants who discontinued the study for any reason were classified as dropouts. The dropout rate was calculated by dividing the number of dropouts by the total number of participants randomised in the study. We further assessed some secondary acceptability outcomes, including (1) mania or hypomania, (2) suicidal ideation and behaviour and (3) any side effects. Regarding side effects, we focused specifically on akathisia and parkinsonism (extrapyramidal events) and analysed these conditions separately. These side effects were defined as adverse events reported during the study. Additionally, we were interested in changes in metabolic and endocrine profiles, as these are potential areas of concern associated with atypical antipsychotics, including (1) body weight; (2) lipid profiles, such as total cholesterol (TC), low-density lipoprotein (LDL) cholesterol and triglycerides (TG); (3) blood sugar levels, such as fasting glucose and glycosylated haemoglobin (HbA1c); and (4) prolactin levels in males and females.

Two independent reviewers (Y-WL and C-WH) evaluated the risk of bias in each included study using the Cochrane Handbook tool.^13^ Any disagreements were resolved through discussion with a third author (P-TT).

### Data synthesis and statistical analysis

For the dose categories, we used the mean dose of lurasidone. In the one-step dose–response meta-analysis, we explored the relationship between lurasidone dosage and outcomes based on previous studies conducted by our team.^11 14 15^ We employed methodologies developed by Greenland and Longnecker^16^ and Orsini *et al*^17^ to account for potential non-linear trends in the data. Specifically, we implemented restricted cubic splines with three knots, placed at fixed percentiles (10th, 50th and 90th),^18^ corresponding to different lurasidone doses. To assess heterogeneity, a variance partition coefficient, an extension of the I^2^ statistic, was used in the single-stage dose–response meta-analysis.^19^ All analyses were performed using the dosresmeta package (V.2.0.1) in R software. Statistical significance was set at p<0.05. Three effect sizes were used in this study. First, we calculated the pre and post changes in depression, anxiety and other assessment scale tools and converted them into standardised mean differences (SMDs) with 95% CIs. Second, we assessed the number of dropouts and events and converted them into risk ratios (RRs) with 95% CIs. Third, we calculated the pre–post changes in metabolic and endocrine data and converted these into mean differences (MDs) with 95% CIs. Finally, we also focused on the number-needed-to-treat (NNT) to observe these outcomes.^13^ For continuous variables, we adopted the method of Kraemer and Kupfer to calculate NNT from the area under the curve, allowing direct calculation from the SMD.^20^

Subsequent to a systematic review, we identified one RCT^21^ involving participants with major depressive disorder (MDD) with mixed features, a condition that often progresses to bipolar disorder.^22^ To assess the treatment efficacy for depressive symptoms in major mood disorders thoroughly, we included this study in our sensitivity analysis.

## Results

The literature search process is depicted in the PRISMA flowchart (online supplemental efigure 1). A full-text review excluded further studies for the reasons detailed in online supplemental eTable 3. Ultimately, five studies were included in the meta-analysis (table 1).^23^,^27^ These five studies encompassed 2032 participants with a mean age of 37.5 years.^23^,^27^ Over half (52.5%, n=1067) were females. Table 1 also lists a study in which participants were diagnosed with MDD with mixed features, which was included for sensitivity analysis. All studies administered lurasidone for 6 weeks, with daily doses ranging from 20 mg to 120 mg.

**Table 1:** Characteristics of included studies

Study	Study design	Diagnosis	Age, year (SD)	Cases number (male/female)	Treatment group, dose range (mean dose)	Treatment duration	Assessment tools	Region
Loebel 2014a	Double-blind, parallel	BDI (DSM-IV-TR)	41.2 (12.5) 41.3 (12.3) 42.0 (12.4)	162 (75/87) 161 (70/91) 162 (64/98)	Placebo Lurasidone 20–60 (32) mg Lurasidone 80–120 (82) mg	6 weeks	MADRS, HAM-A, CGI-BP, SDS, Q-LES-Q-SF	Africa, Asia, Europe, USA
Loebel 2014b^1^	Double-blind, parallel	BDI (DSM-IV-TR)	42.6 (11.8) 41.0 (11.5)	161 (85/76) 179 (93/86)	Placebo Lurasidone 20–120 (66) mg	6 weeks	MADRS, HAM-A, CGI-BP, SDS, Q-LES-Q-SF	Africa, Asia, Europe, USA
Suppes 2016a^1^	Double-blind, parallel	BDI (DSM-IV-TR)	44.1 (12.0) 43.1 (11.9)	166 (73/93) 176 (85/91)	Placebo Lurasidone 20–120 (65) mg	6 weeks	MADRS, HAM-A, CGI-BP, SDS, Q-LES-Q-SF	Asia, Europe, South America, USA
DelBello 2017	Double-blind, parallel	BDI (DSM-5)	14.3 (2.0) 14.2 (2.2)	170 (87/83) 173 (88/85)	Placebo Lurasidone 20–80 (33) mg	6 weeks	CDCS-R, PARS, CGI-BP, Q-LES-Q	Asia, Europe, Mexico, USA
Kato 2020	Double-blind, parallel	BDI (DSM-5)	41.3 (12.6) 42.6 (12.9) 43.2 (12.8)	171 (77/94) 182 (87/95) 169 (81/88)	Placebo Lurasidone 20–60 (36) mg Lurasidone 80–120 (85) mg	6 weeks	MADRS, HAM-A, CGI-BP, SDS	Asia, Europe
Suppes 2016b	Double-blind, parallel	MDD-MF (DSM-IV-TR)	46.4 (12.0) 43.6 (12.1)	100 (28/72) 109 (36/73)	Placebo Lurasidone 20–60 (36) mg	6 weeks	MADRS, HAM-A, CGI-S, SDS	Europe, USA

BDI, bipolar I disorder; CDCS-R, Children’s Depression Rating Scale-revised; CGI-BP, clinical global impression - bipolar disorder severity; CGI-S, clinical global impression - severity; DSM-5, Diagnostic and Statistical Manual of Mental Disorders, fifth edition; DSM-IV-TR, Diagnostic and Statistical Manual of Mental Disorders, fourth edition, text revised; HAM-A, Hamilton Anxiety Rating Scale; MADRS, Montgomery-Asberg Depression Rating Scale; MDD-MF, major depressive disorder with mixed features; PARS, Paediatric Anxiety Rating Scale; Q-LES-Q-SF, Quality of Life Enjoyment and Satisfaction Questionnaire–short form; SDS, Sheehan Disability Scale.

**Figure 1:**
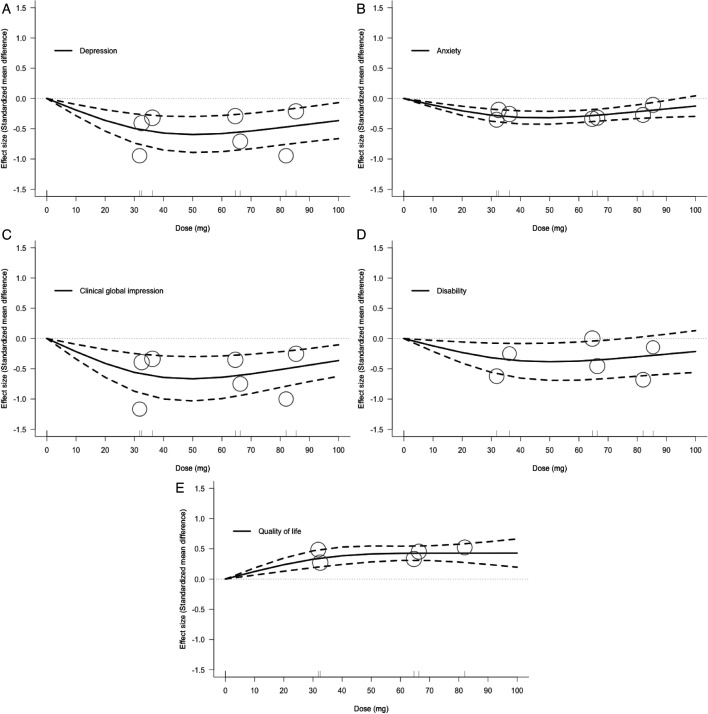
Dose–response relationship between daily lurasidone doses and efficacy. (**A**) Depression; (**B**) anxiety; (**C**) clinical global impression; (**D**) disability; (**E**) quality of life. Solid line: pooled point estimates; dotted line: 95% CI; short vertical lines on the x-axis: lurasidone dose of the included studies; open circles: outcome markers for all included studies, whose size represents the reciprocal of the SE of the effect size.

The 40–60 mg dose of lurasidone was associated with optimal efficacy for most outcomes (figure 1 and table 2). For depressive symptoms, lurasidone achieved the best improvement effect in the range of 40–60 mg and reached the peak effect at 50 mg (SMD −0.60, 95% CI −0.30, –0.89; figure 1A). A similar trend was observed for anxiety symptoms in the dose–response analysis, with the 40–60 mg range showing the best effect and reaching peak effect at 50 mg (SMD −0.32, 95% CI −0.21, –0.42; figure 1B). CGI score improvement (50 mg: SMD −0.67, 95% CI −0.30, –1.03; figure 1C) and disability reduction (50 mg: SMD −0.38, 95% CI −0.08, –0.69; figure 1D) were also optimal in the 40–60 mg range. For QOL, the curve showed that the QOL improved as the lurasidone dose increased to the 40–60 mg range and then plateaued after 60 mg (60 mg: SMD 0.43, 95% CI 0.31, 0.54; 100 mg: SMD 0.43, 95% CI 0.20, 0.66; figure 1E). Additionally, a sensitivity analysis that incorporated MDD with mixed features found that a dose range of 40–60 mg was also effective in treating depressive symptoms (online supplemental eTable 4).

**Table 2:** Estimated effect sizes from dose–response meta-analysis

Outcome	Lurasidone dose									
	10 mg	20 mg	30 mg	40 mg	50 mg	60 mg	70 mg	80 mg	90 mg	100 mg
Efficacy (SMD)										
Depression	−0.19 (-0.28,–0.10)*	−0.36 (-0.54,–0.19)*	−0.50 (-0.74,–0.25)*	−0.57 (-0.85,–0.29)*	−0.60 (-0.89,–0.30)*	−0.58 (-0.88,–0.28)*	−0.54 (-0.83,–0.25)*	−0.48 (-0.77,–0.19)*	−0.42 (-0.71,–0.13)*	−0.37 (-0.66,–0.07)*
Anxiety	−0.11 (-0.15,–0.07)*	−0.20 (-0.28,–0.13)*	−0.28 (-0.38,–0.18)*	−0.31 (-0.42,–0.21)*	−0.32 (-0.42,–0.21)*	−0.30 (-0.40,–0.20)*	−0.26 (-0.36,–0.16)*	−0.22 (-0.33,–0.10)*	−0.17 (-0.31,–0.03)*	−0.13 (-0.30,0.04)
CGI	−0.22 (-0.34,–0.10)*	−0.41 (-0.64,–0.18)*	−0.56 (-0.87,–0.25)*	−0.64 (-1.00,–0.29)*	−0.67 (-1.03,–0.30)*	−0.64 (-0.99,–0.29)*	−0.59 (-0.91,–0.26)*	−0.51 (-0.81,–0.22)*	−0.44 (-0.71,–0.17)*	−0.36 (-0.63,–0.10)*
Disability	−0.12 (-0.21,–0.03)*	−0.23 (-0.41,–0.06)*	−0.32 (-0.56,–0.08)*	−0.37 (-0.65,–0.08)*	−0.38 (-0.69,–0.08)*	−0.37 (-0.69,–0.06)*	−0.34 (-0.66,–0.02)*	−0.30 (-0.62,0.02)	−0.26 (-0.59,0.07)	−0.21 (-0.56,0.13)
Quality of life	0.12 (0.07,0.18)*	0.24 (0.13,0.35)*	0.33 (0.19,0.47)*	0.38 (0.24,0.53)*	0.41 (0.28,0.55)*	0.43 (0.31,0.54)*	0.43 (0.31,0.55)*	0.43 (0.28,0.58)*	0.43 (0.24,0.62)*	0.43 (0.20,0.66)*
Acceptability (RR)										
Dropout	0.97 (0.89,1.05)	0.94 (0.80,1.1)	0.92 (0.75,1.14)	0.92 (0.73,1.16)	0.94 (0.75,1.17)	0.96 (0.78,1.18)	1.00 (0.82,1.21)	1.04 (0.84,1.28)	1.08 (0.83,1.40)	1.12 (0.82,1.54)
Mania	1.1 (0.89,1.35)	1.17 (0.79,1.74)	1.21 (0.71,2.04)	1.16 (0.65,2.10)	1.06 (0.58,1.95)	0.93 (0.49,1.75)	0.79 (0.39,1.60)	0.66 (0.28,1.52)	0.55 (0.20,1.51)	0.46 (0.14,1.54)
Suicide	0.98 (0.87,1.10)	0.96 (0.77,1.20)	0.95 (0.71,1.27)	0.94 (0.69,1.30)	0.95 (0.69,1.30)	0.96 (0.72,1.29)	0.97 (0.74,1.29)	0.99 (0.74,1.34)	1.01 (0.71,1.43)	1.03 (0.67,1.57)
Any side effect	1.04 (1.00,1.07)*	1.07 (1.01,1.15)*	1.11 (1.01,1.2)*	1.13 (1.03,1.24)*	1.15 (1.05,1.25)*	1.16 (1.07,1.26)*	1.16 (1.07,1.26)*	1.17 (1.06,1.28)*	1.17 (1.04,1.32)*	1.18 (1.02,1.36)*
Akathisia	1.19 (1.02,1.38)*	1.40 (1.06,1.86)*	1.63 (1.12,2.38)*	1.88 (1.24,2.86)*	2.13 (1.40,3.24)*	2.39 (1.60,3.56)*	2.66 (1.82,3.90)*	2.96 (2.00,4.37)*	3.28 (2.13,5.06)*	3.64 (2.2,6.02)*
Parkinsonism	1.00 (0.83,1.21)	1.01 (0.71,1.44)	1.06 (0.67,1.67)	1.15 (0.71,1.88)	1.31 (0.83,2.06)	1.53 (1.02,2.28)*	1.82 (1.25,2.65)*	2.20 (1.43,3.39)*	2.66 (1.52,4.64)*	3.21 (1.57,6.56)*
Metabolism / Endocrinology (MD)										
Weight (kg)	0.15 (0.07,0.24)*	0.28 (0.13,0.44)*	0.37 (0.16,0.57)*	0.38 (0.16,0.60)*	0.33 (0.11,0.55)*	0.23 (0.00,0.46)	0.10 (-0.18,0.38)	−0.05 (-0.41,0.31)	−0.20 (-0.67,0.26)	−0.36 (-0.93,0.22)
TC (mg/dL)	0.07 (-1.64,1.78)	0.12 (-3.02,3.26)	0.13 (-3.91,4.16)	0.07 (-4.11,4.26)	−0.04 (-3.75,3.68)	−0.19 (-3.24,2.87)	−0.37 (-3.31,2.57)	−0.56 (-4.43,3.30)	−0.76 (-6.17,4.65)	−0.96 (-8.13,6.22)
LDL (mg/dL)	−0.04 (-1.03,0.94)	−0.10 (-1.93,1.73)	−0.19 (-2.60,2.23)	−0.31 (-2.95,2.32)	−0.48 (-3.05,2.09)	−0.68 (-3.07,1.72)	−0.89 (-3.24,1.45)	−1.12 (-3.72,1.47)	−1.35 (-4.48,1.78)	−1.58 (-5.42,2.26)
TG (mg/dL)	−2.44 (-6.34,1.46)	−4.34 (-11.44,2.75)	−5.18 (-14.10,3.74)	−4.50 (-13.45,4.46)	−2.37 (-10.27,5.52)	0.83 (-6.84,8.50)	4.76 (-5.39,14.90)	9.05 (-5.63,23.74)	13.42 (-6.61,33.45)	17.79 (-7.87,43.45)
Glucose (mg/dL)	0.05 (-0.48,0.58)	0.13 (-0.86,1.12)	0.28 (-1.02,1.59)	0.53 (-0.89,1.95)	0.87 (-0.53,2.27)	1.27 (-0.07,2.62)	1.73 (0.34,3.11)*	2.20 (0.60,3.81)*	2.68 (0.72,4.65)*	3.16 (0.76,5.57)*
HbA1c (%)	0.01 (0.00,0.02)	0.01 (-0.01,0.04)	0.02 (-0.01,0.05)	0.02 (-0.01,0.06)	0.03 (-0.01,0.06)	0.03 (-0.01,0.07)	0.03 (-0.01,0.07)	0.03 (-0.01,0.07)	0.03 (-0.02,0.08)	0.03 (-0.02,0.09)
Prolactin, male (ng/mL)	0.71 (-0.03,1.46)	1.41 (0.04,2.77)*	2.06 (0.31,3.82)*	2.66 (0.85,4.48)*	3.21 (1.59,4.84)*	3.72 (2.37,5.08)*	4.21 (2.88,5.53)*	4.68 (2.94,6.42)*	5.15 (2.73,7.56)*	5.61 (2.42,8.81)*
Prolactin, female (ng/mL)	2.02 (0.78,3.26)*	3.85 (1.55,6.15)*	5.31 (2.29,8.33)*	6.23 (2.96,9.50)*	6.64 (3.50,9.78)*	6.67 (3.81,9.53)*	6.45 (3.71,9.19)*	6.09 (3.04,9.14)*	5.71 (1.97,9.46)*	5.33 (0.67,10.00)*

An asterisk with grey background indicates statistical significance.

CGI, clinical global impression; HbA1c, glycosylated haemoglobin; LDL, low-density lipoprotein; MD, mean difference; RR, risk ratio; SMD, standardised mean difference; TC, total cholesterol; TG, triglyceride.

**Figure 2:**
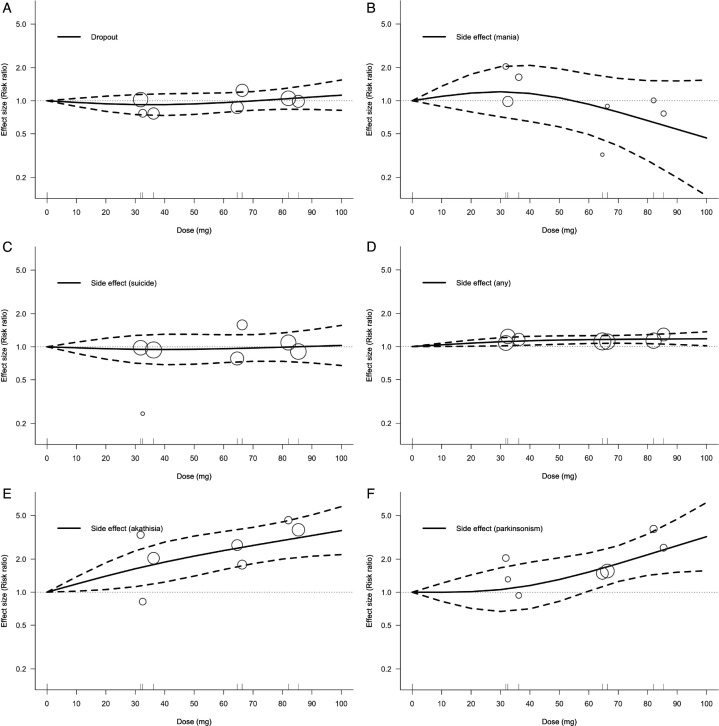
Dose–response relationship between daily lurasidone doses and acceptability. (**A**) Dropout; (**B**) mania switch; (**C**) suicidality; (**D**) any side effect; (**E**) akathisia; (**F**) Parkinsonism. Solid line: pooled point estimates; dotted line: 95% CI; short vertical lines on the x-axis: lurasidone dose of the included studies; open circles: outcome markers for all included studies, whose size represents the reciprocal of the SE of the effect size.

Analysis for acceptability showed no significantly increased risk of dropout observed at any dose of lurasidone (40 mg: RR 0.92, 95% CI 0.73, 1.16; 80 mg: RR 1.04, 95% CI 0.84, 1.28; figure 2A). Similarly, for the occurrence of mania or hypomania switch (40 mg: RR 1.16, 95% CI 0.65, 2.10; 80 mg: RR 0.66, 95% CI 0.28, 1.52; figure 2B) or suicidal ideation or behaviour (40 mg: RR 0.94, 95% CI 0.69, 1.30; 80 mg: RR 0.99, 95% CI 0.74, 1.34; figure 2C), no relationship between these adverse events and dose were observed. However, when examining the occurrence of any side effects, an increased risk was observed with increasing dose (40 mg: RR 1.13, 95% CI 1.03, 1.24; 80 mg: RR 1.17, 95% CI 1.06, 1.28; figure 2D). Moreover, the side effect subcategories also showed that higher lurasidone doses were associated with an increased risk of akathisia (40 mg: RR 1.88, 95% CI 1.24, 2.86; 80 mg: RR 2.96, 95% CI 2.00, 4.37; figure 2E) and parkinsonism (40 mg: RR 1.15, 95% CI 0.71, 1.88; 80 mg: RR 2.20, 95% CI 1.43, 3.39; figure 2F).

**Figure 3:**
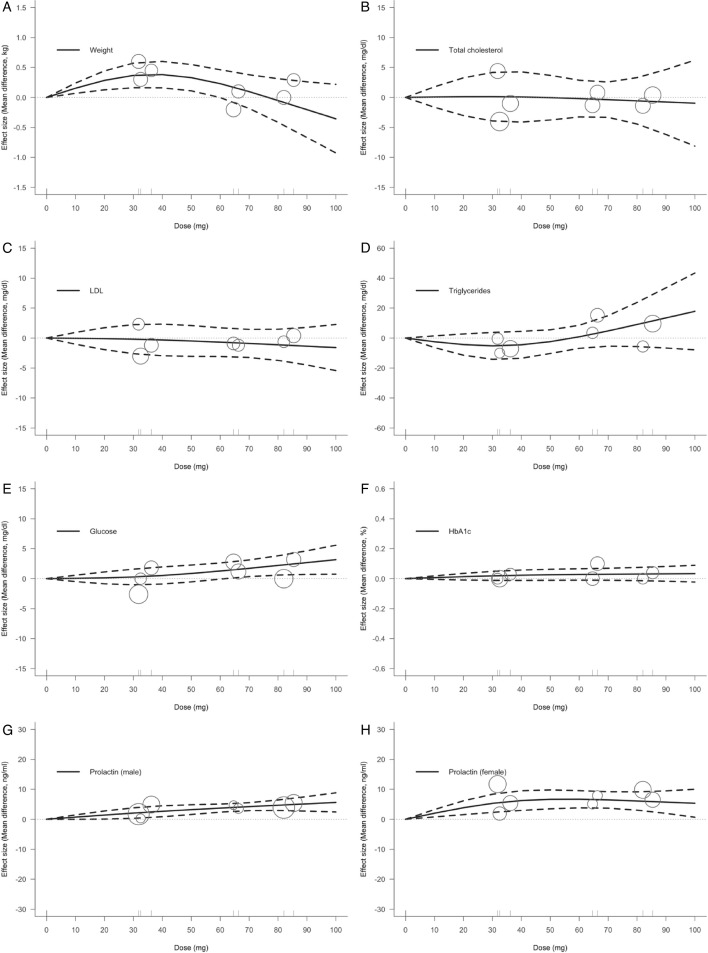
Dose–response relationship between daily lurasidone doses and metabolic or endocrine profiles. (**A**) Body weight; (**B**) total cholesterol; (**C**) low-density lipoprotein; (**D**) triglycerides; (**E**) fasting glucose; (**F**) glycosylated haemoglobin; (**G**) prolactin levels (male); (**H**) prolactin levels (female). Solid line: pooled point estimates; dotted line: 95% CI; short vertical lines on the x-axis: lurasidone dose of the included studies; open circles: outcome markers for all included studies, whose size represents the reciprocal of the SE of the effect size.

Different target outcomes exhibited different relationships between the dose and metabolic or endocrine characteristics (figure 3). The relationship between dose and weight was associated with a convex shape (figure 3A). Using the 40 mg dose as a threshold, body weight increased progressively with increasing doses of lurasidone (MD 0.38 kg, 95% CI 0.16, 0.60). When the dose exceeded 60 mg (MD 0.23 kg, 95% CI 0.00, 0.46), further increases in the dose had no significant effect on body weight. Second, regarding blood lipid levels, no specific relationships of lurasidone treatment dose with TC (figure 3B), LDL (figure 3C) or TG (figure 3D) levels. Third, the effects of lurasidone on glucose metabolism were manifested in fasting blood glucose and HbA1c levels. Fasting blood glucose values tended to increase as the dose increased; however, statistical significance was only observed above 70 mg (MD 1.73 mg/dL, 95% CI 0.34, 3.11; figure 3E). However, this study did not find a significant dose–response relationship between lurasidone and HbA1c (figure 3F). Fourth, a positive dose–effect relationship was seen between prolactin levels and lurasidone dose in males (40 mg: MD 2.66 ng/mL, 95% CI 0.85, 4.48; 80 mg: MD 4.68 ng/mL, 95% CI 2.94, 6.42; figure 3G) or females (40 mg: MD 6.23 ng/mL, 95% CI 2.96, 9.50; 80 mg: MD 6.09 ng/mL, 95% CI 3.04, 9.14; figure 3H).

The estimated NNT for these results are listed in online supplemental eTable 5. The detailed quality assessments, conducted using the Cochrane Risk-of-Bias 2 tool, are shown in online supplemental eTable 6 and online supplemental efigure 2. Of the five included trials, four were judged to have a low risk-of-bias^23 24 26 27^ while the remaining study had some concerns for risk-of-bias.^25^ The variance partition coefficients for the primary outcomes are shown in online supplemental efigure 3.

## Discussion

Based on the SMD values, the 40–60 mg dose range of lurasidone had optimal efficacy across multiple outcomes, including a reduction of depression, anxiety, CGI scores and disability. Regarding acceptability, although dose escalation was associated with an increased incidence of side effects (any side effect, akathisia and parkinsonism), no significant dose-dependent associations were observed for the main outcomes, such as dropout rates, switch from depression to mania or occurrence of suicidal events. Furthermore, the effects of lurasidone on metabolic syndrome and the endocrine system vary based on specific outcomes. Weight gain was significantly associated with doses below 60 mg, whereas increased fasting blood glucose levels were associated with doses above 70 mg. No significant associations between the dose and serum lipid (including TC, LDL and TG levels) or HbA1c levels were found. Prolactin levels increased consistently with increasing doses, in both male and female patients.

Our meta-analysis showed optimal efficacy between doses of 40 mg and 60 mg. We have postulated several hypotheses regarding the underlying mechanisms. Current evidence indicates that 5-hydroxytryptamine 7 (5-HT7) receptor antagonism may contribute to positive effects on mood and memory.^28 29^ On the other hand, histamine-1, alpha-1 and alpha-2A receptor antagonism may lead to depressant effects on the central nervous system, which could cause potential cognitive impairment.^30 31^ Lurasidone is a strong 5-HT7 receptor antagonist, but it also has weak antagonism effects on the histamine-1, alpha-1 and alpha-2A receptors.^30^ While antidepressant effects may be preserved at lower doses of the drug, higher doses could compromise cognitive function, leading to attenuated antidepressant efficacy. Additionally, a previous study ranked the NNT to decrease depressive symptoms in bipolar disorder as follows: olanzapine plus fluoxetine (NNT: 4), lurasidone (NNT: 5), quetiapine (NNT: 6) and lamotrigine (NNT: 6).^32^ However, when lurasidone was set within the specific dose range of 40–60 mg, the present study showed a pooled NNT of 3 (online supplemental eTable 5), suggesting that appropriate drug doses may have better therapeutic effects than those of typical doses (20–120 mg).^33^

In terms of acceptability, this meta-analysis found no significant association between lurasidone dosage and the incidence of critical issues, such as dropout, mania or suicide. These findings suggest that increasing doses of lurasidone remain relatively safe, mitigating concerns about their usage in clinical practice. However, our results still indicate that, with increasing dose, the risk of side effects, such as akathisia or parkinsonism, do increase.^23^,^27^ This highlights the continued need for clinicians to be aware of these non-urgent adverse effects when adjusting lurasidone dosage. Additionally, we observed a trend of protective efficacy against manic switch at daily doses above 50–60 mg (figure 2B). This may be explained by lurasidone’s role as a full antagonist of the dopamine D2 receptor.^34 35^

Lurasidone has three broad categories of metabolic and endocrine effects. First, certain dose ranges significantly increase the risk of adverse effects, such as weight gain. Below 60 mg, weight increased significantly (figure 3A). Interestingly, a trend towards weight reduction was observed at higher doses of lurasidone (greater than 70–80 mg). This effect may be attributed to lurasidone’s 5-HT7 receptor inverse agonism, which suppresses adenosine monophosphate-activated protein kinase signalling in a concentration-dependent manner, thereby mitigating the risk of weight gain as compared with other antipsychotics.^36^ Another outcome measure was an increase in fasting blood glucose levels. Our findings suggested a significant risk at doses above 70 mg (figure 3E), although HbA1c was not associated with a dose-related risk (figure 3F). This discrepancy may be attributed to the fact that HbA1c typically reflects average blood glucose levels over a 3-month period,^37^ whereas the treatment duration of the included studies was only 6 weeks, possibly not allowing sufficient time for changes in HbA1c levels to manifest. Taken together, although a previous network meta-analysis suggested that lurasidone may pose minimal risks to glucose concentrations,^38^ caution is warranted regarding the potential effects of high doses of lurasidone on glucose metabolism. Second, certain dose categories are positively associated with risk, such as increased prolactin levels in males and females. Our findings support those of previous reviews showing that lurasidone is comparable to many known antipsychotics (eg, risperidone and haloperidol) in inducing increased prolactin levels.^39^ Third, doses and certain risks, such as TC, TG, and LDL cholesterol, were not related.

The US Food and Drug Administration (FDA) recommends a dose of lurasidone of 20–120 mg/day for bipolar disorder and 40–160 mg/day for schizophrenia.^33^ The difference in the lower and upper thresholds of doses prescribed for the two disorders may reflect the drug’s distinct actions on certain receptors (dopamine, 5-HT and alpha), which mediate its antipsychotic and antidepressant effects.^34^ Research has shown that at least 60% dopamine occupancy is required to achieve clinically responsive antipsychotic efficacy when treating schizophrenia,^33^ and higher doses of lurasidone (over 80 mg) achieves better dopamine D2 receptor occupancy levels.^40^ In contrast, antidepressant mechanisms are affected by more complex interactions (5-HT and alpha),^34^ and no direct positive correlation between dose and efficacy exists. Collectively, based on the FDA-approved range of 20–120 mg/day and the dose–response curve demonstrated in this study, we inform clinicians that the optimal therapeutic efficacy of lurasidone for treating bipolar depression is generally observed at 40–60 mg/day. However, clinicians should consider individual patient acceptability and metabolic/endocrine status when determining the appropriate individualised dose. Additionally, our sensitivity analysis, including MDD with mixed features, showed that lurasidone might also exhibit antidepressant efficacy (table 2 and online supplemental eTable 4). Some post-hoc analyses from RCTs included in this study found that lurasidone was equally effective in treating bipolar depression with mixed features.^34 41 42^ These findings may suggest a therapeutic role for lurasidone in mood disorders with mixed features. One study even suggested that lurasidone may outperform other antipsychotics (ziprasidone and olanzapine).^43^ However, this conclusion has not been substantiated by corresponding RCTs and requires further investigation.

This study also had some limitations. First, a limited number of RCTs have investigated the use of lurasidone in bipolar disorder, and only five RCTs that met the inclusion criteria were included in this study. Nonetheless, these included RCTs primarily originated from the drug development phase and were mostly assessed as having a low risk-of-bias; hence, the overall findings still provide valuable insights. Second, due to the limited number of included studies, independent subgroup analysis for different age groups was not possible. For instance, one study only involved adolescent patients,^6^ and no studies specifically focused on older patients with bipolar disorder.^44^ Third, QTc prolongation is an important side effect of antipsychotics.^39^ One review concluded that lurasidone does not affect the QTc interval.^34^ However, we did not include these results in this meta-analysis because only three studies reported this outcome,^23 24 27^ and they only reported changes in the mean, which was not sufficient for a meaningful analysis. Finally, the studies included in this meta-analysis involved only participants with bipolar I depression, and there were no studies involving participants with bipolar II depression. However, the two types of bipolar depression differ in their epidemiology, clinical course, genetics and response to treatment.^45^ Therefore, we advocate for further RCTs in the future to focus on the use of lurasidone in treating bipolar II disorder.

### Conclusions and clinical implications

This study included five RCTs and used a dose–response meta-analysis to establish dosing recommendations for lurasidone for the treatment of bipolar depression. In terms of efficacy, a daily dose of 40–60 mg showed optimal effects in improving depressive and anxiety symptoms, CGI score and disability. In terms of acceptability, although increasing the dose increased the risk of side effects, no dose–effect relationship was seen for major adverse outcomes, such as dropout incidence, manic switch and suicidality. Therefore, current evidence suggested that the clinical dose target for lurasidone may be primarily in the 40–60 mg range, which can be adjusted based on individual patient conditions.

## Supplementary material

10.1136/bmjment-2024-301165 online supplemental file 1

## Data Availability

Data are available upon reasonable request.
