# Supplementary material for: Efficacy and acceptability of lurasidone for bipolar depression: a systematic review and dose–response meta-analysis
Source: BMJ Ment Health. 2024 Nov 18;27(1):e301165. doi: 10.1136/bmjment-2024-301165 (PMC11574478; doi:10.1136/bmjment-2024-301165)
Supplement: online supplemental file 1 [file bmjment-27-1-s001.pdf]

Supplemental data

Dose-response association of lurasidone in the treatment of bipolar depression: A systematic review and meta-analysis

Running Title: lurasidone for bipolar depression

Yu-Wei Lin, MD[a], Yang-Chieh Brian Chen, MD[a], Kuo-Chuan Hung, MD[b], Chih-Sung Liang, MD[c,d], Ping-Tao Tseng, MD, PhD[e,f,g,h], Andre F. Carvalho, MD, PhD[i], Eduard Vieta, MD, PhD[j], Marco Solmi, MD, PhD[k,l,m,n,o], Edward Chia-Cheng Lai, PhD[p], Pao-Yen Lin, MD, PhD[a], Chih-Wei Hsu, MD[a,*], Yu-Kang Tu, PhD[q,r]

[a] Department of Psychiatry, Kaohsiung Chang Gung Memorial Hospital and Chang Gung University College of Medicine, Kaohsiung, Taiwan
[b] Department of Anesthesiology, Chi Mei Medical Center, Tainan, Taiwan
[c] Department of Psychiatry, Beitou Branch, Tri-Service General Hospital, National Defense Medical Center, Taipei, Taiwan
[d] Department of Psychiatry, National Defense Medical Center, Taipei, Taiwan
[e] Prospect Clinic for Otorhinolaryngology & Neurology, Kaohsiung, Taiwan
[f] Institute of Biomedical Sciences, National Sun Yat-sen University, Kaohsiung, Taiwan
[g] Department of Psychology, College of Medical and Health Science, Asia University, Taichung, Taiwan
[h] Institute of Precision Medicine, National Sun Yat-sen University, Kaohsiung City, Taiwan
[i] Innovation in Mental and Physical Health and Clinical Treatment (IMPACT) Strategic Research Centre, School of Medicine, Barwon Health, Deakin University, Geelong, VIC, Australia
[j] Bipolar and Depressive Disorders Unit, Hospital Clinic, IDIBAPS, CIBERSAM, University of Barcelona, Barcelona, Catalonia, Spain
[k] Department of Psychiatry, University of Ottawa, Ottawa, Canada
[l] Department of Mental Health, The Ottawa Hospital, Ottawa, Canada
[m] Ottawa Hospital Research Institute (OHRI), Clinical Epidemiology Program, University of Ottawa, Ottawa, Canada
[n] School of Epidemiology and Public Health, Faculty of Medicine, University of Ottawa, Ottawa, Canada
[o] Department of Child and Adolescent Psychiatry, Charité Universitätsmedizin, Berlin, Germany
[p] School of Pharmacy, Institute of Clinical Pharmacy and Pharmaceutical Sciences, College of Medicine, National Cheng Kung University, Tainan, Taiwan
[q] Institute of Epidemiology and Preventive Medicine, National Taiwan University College of Public Health, Taipei, Taiwan
[r] Department of Dentistry, National Taiwan University Hospital, Taipei, Taiwan

*** Corresponding author:**
Chih-Wei Hsu
Department of Psychiatry, Kaohsiung Chang Gung Memorial Hospital
No. 123, Dapi Road, Niaosong District, Kaohsiung City 833, Taiwan
Telephone number: 886-7-7317123 ext. 8753
Fax number: 886-7-7326817
E-mail address: harwicacademia@gmail.com

**Content**	**Page**
eTable 1. PRISMA Checklist	1-2
eTable 2. Detailed search strategy	3
eTable 3. Excluded studies and reason	4
eTable 4. Sensitivity analysis including major depressive disorder with mixed features	5
eTable 5. Number needed to harm/treat from dose-response meta-analysis	6
eTable 6. Detailed quality assessment of included studies using Cochrane risk of bias 2 tool	7
eFigure 1. Flowchart of study selection	8
eFigure 2. Summary of quality assessment of included studies using Cochrane risk of bias 2 tool	9
eFigure 3. Variation partition coefficients of primary outcomes	10-11
References	12

eTable 1. PRISMA Checklist

Section and Topic	#	Checklist item	Location
**TITLE**			
Title	1	Identify the report as a systematic review.	Title page
**ABSTRACT**			
Abstract	2	See the PRISMA 2020 for Abstracts checklist.	3
**INTRODUCTION**			
Rationale	3	Describe the rationale for the review in the context of existing knowledge.	1
Objectives	4	Provide an explicit statement of the objective(s) or question(s) the review addresses.	2
**METHODS**			
Eligibility criteria	5	Specify the inclusion and exclusion criteria for the review and how studies were grouped for the syntheses.	3-4
Information sources	6	Specify all databases, registers, websites, organisations, reference lists and other sources searched or consulted to identify studies. Specify the date when each source was last searched or consulted.	3
Search strategy	7	Present the full search strategies for all databases, registers and websites, including any filters and limits used.	3, eTable 2
Selection process	8	Specify the methods used to decide whether a study met the inclusion criteria of the review, including how many reviewers screened each record and each report retrieved, whether they worked independently, and if applicable, details of automation tools used in the process.	3-4
Data collection process	9	Specify the methods used to collect data from reports, including how many reviewers collected data from each report, whether they worked independently, any processes for obtaining or confirming data from study investigators, and if applicable, details of automation tools used in the process.	4
Data items	10a	List and define all outcomes for which data were sought. Specify whether all results that were compatible with each outcome domain in each study were sought (e.g., for all measures, time points, analyses), and if not, the methods used to decide which results to collect.	4
	10b	List and define all other variables for which data were sought (e.g., participant and intervention characteristics, funding sources). Describe any assumptions made about any missing or unclear information.	4
Study risk of bias assessment	11	Specify the methods used to assess risk of bias in the included studies, including details of the tool(s) used, how many reviewers assessed each study and whether they worked independently, and if applicable, details of automation tools used in the process.	4-5
Effect measures	12	Specify for each outcome the effect measure(s) (e.g., risk ratio, mean difference) used in the synthesis or presentation of results.	5
Synthesis methods	13a	Describe the processes used to decide which studies were eligible for each synthesis (e.g., tabulating the study intervention characteristics and comparing against the planned groups for each synthesis (item #5)).	4
	13b	Describe any methods required to prepare the data for presentation or synthesis, such as handling of missing summary statistics, or data conversions.	5
	13c	Describe any methods used to tabulate or visually display results of individual studies and syntheses.	Table 2
	13d	Describe any methods used to synthesize results and provide a rationale for the choice(s). If meta-analysis was performed, describe the model(s), method(s) to identify the presence and extent of statistical heterogeneity, and software package(s) used.	5
	13e	Describe any methods used to explore possible causes of heterogeneity among study results (e.g., subgroup analysis, meta-regression).	5
	13f	Describe any sensitivity analyses conducted to assess robustness of the synthesized results.	5, eTable 4
Reporting bias assessment	14	Describe any methods used to assess risk of bias due to missing results in a synthesis (arising from reporting biases).	NA
Certainty assessment	15	Describe any methods used to assess certainty (or confidence) in the body of evidence for an outcome.	NA
**RESULTS**			
Study selection	16a	Describe the results of the search and selection process, from the number of records identified in the search to the number of studies included in the review, ideally using a flow diagram.	6, eFigure 1
	16b	Cite studies that might appear to meet the inclusion criteria, but which were excluded, and explain why they were excluded.	eTable 3
Study characteristics	17	Cite each included study and present its characteristics.	6, Table 1
Risk of bias	18	Present assessments of risk of bias for each included study.	eTable 6, eFigure 2
Results of individual studies	19	For all outcomes, present, for each study: (a) summary statistics for each group (where appropriate) and (b) an effect estimates and its precision (e.g., confidence/credible interval), ideally using structured tables or plots.	6-7, Figure 1-3

Results of syntheses	20a	For each synthesis, briefly summarise the characteristics and risk of bias among contributing studies.	7, eTable 6, eFigure 2
	20b	Present results of all statistical syntheses conducted. If meta-analysis was done, present for each the summary estimate and its precision (e.g., confidence/credible interval) and measures of statistical heterogeneity. If comparing groups, describe the direction of the effect.	6-7, Figure 1-3, eFigure 3
	20c	Present results of all investigations of possible causes of heterogeneity among study results.	eFigure 3
	20d	Present results of all sensitivity analyses conducted to assess the robustness of the synthesized results.	7, eTable 4
Reporting biases	21	Present assessments of risk of bias due to missing results (arising from reporting biases) for each synthesis assessed.	eTable 6, eFigure 2
Certainty of evidence	22	Present assessments of certainty (or confidence) in the body of evidence for each outcome assessed.	NA
**DISCUSSION**			
Discussion	23a	Provide a general interpretation of the results in the context of other evidence.	8
	23b	Discuss any limitations of the evidence included in the review.	10
	23c	Discuss any limitations of the review processes used.	10
	23d	Discuss implications of the results for practice, policy, and future research.	10-11
**OTHER INFORMATION**			
Registration and protocol	24a	Provide registration information for the review, including register name and registration number, or state that the review was not registered.	3
	24b	Indicate where the review protocol can be accessed, or state that a protocol was not prepared.	3
	24c	Describe and explain any amendments to information provided at registration or in the protocol.	3
Support	25	Describe sources of financial or non-financial support for the review, and the role of the funders or sponsors in the review.	12
Competing interests	26	Declare any competing interests of review authors.	12
Availability of data, code and other materials	27	Report which of the following are publicly available and where they can be found: template data collection forms; data extracted from included studies; data used for all analyses; analytic code; any other materials used in the review.	12

eTable 2. Keywords and search results in different databases

Database	Keyword	Filter	Date	Results
PubMed	("lurasidone" OR "SM-13496") AND ("depress*" OR "bipolar" OR "affective" OR "mood")	Not applied	1 August 2024	376
Embase	("lurasidone" OR "SM-13496") AND ("depress*" OR "bipolar" OR "affective" OR "mood")	Title Abstract Keyword	1 August 2024	729
Cochrane CENTRAL	("lurasidone" OR "SM-13496") AND ("depress*" OR "bipolar" OR "affective" OR "mood")	Title Abstract Keyword	1 August 2024	318
ScienceDirect	("lurasidone" OR "SM-13496") AND ("depress" OR "bipolar" OR "affective" OR "mood")	Title Abstract Keyword	1 August 2024	141
ClinicalTrials.gov	("lurasidone" OR "SM-13496") AND ("depress*" OR "bipolar" OR "affective" OR "mood")	Condition or disease Intervention/ Treatment	1 August 2024	35

Keyword adjusted as below in ScienceDirect due to wildcard* was not applicable: ("lurasidone" OR "SM-13496") AND ("depress" OR "bipolar" OR "affective" OR "mood"); Cochrane CENTRAL and ClinicalTrials.gov were classified as registries in the PRISMA flowchart (Figure 1).

Gray literature N = 150

Use the keywords ("lurasidone" OR "SM-13496") AND ("depress*" OR "bipolar" OR "affective" OR "mood"), to search the following gray literature.

1. Airiti Library (https://www.airitilibrary.com/Home/Index), n = 9
2. CADTH checklist (https://www.cadth.ca/), n = 0
3. ISRCTN Registry (https://www.isrctn.com/), n = 2
4. OAIster (https://oaister.worldcat.org/), n = 45
5. World Health Organization International Clinical Trials Registry Platform (ICTRP) (https://trialsearch.who.int/Default.aspx), n = 94

eTable 3. Excluded studies and reasons

Reasons	Reference
**Not randomized controlled trials**	Forester, B. P., Sajatovic, M., Tsai, J., Pikalov, A., Cucchiaro, J., & Loebel, A. (2018). Safety and Effectiveness of Long-Term Treatment with Lurasidone in Older Adults with Bipolar Depression: Post-Hoc Analysis of a 6-Month, Open-Label Study. The American journal of geriatric psychiatry: official journal of the American Association for Geriatric Psychiatry, 26(2), 150–159.<br><br>Singh, M. K., Pikalov, A., Siu, C., Tocco, M., & Loebel, A. (2020). Lurasidone in Children and Adolescents with Bipolar Depression Presenting with Mixed (Subsyndromal Hypomanic) Features: Post Hoc Analysis of a Randomized Placebo-Controlled Trial. Journal of child and adolescent psychopharmacology, 30(10), 590–598.
**Participants overlap with other studies**	Ketter TA, Sarma K, Silva R, Kroger H, Cucchiaro J, Loebel A. LURASIDONE IN THE LONG-TERM TREATMENT OF PATIENTS WITH BIPOLAR DISORDER: A 24-WEEK OPEN-LABEL EXTENSION STUDY. Depress Anxiety. 2016 May;33(5):424-34.<br><br>DelBello MP, Tocco M, Pikalov A, Deng L, Goldman R. Tolerability, Safety, and Effectiveness of Two Years of Treatment with Lurasidone in Children and Adolescents with Bipolar Depression. J Child Adolesc Psychopharmacol. 2021 Sep;31(7):494-503.<br><br>Higuchi, T., Kato, T., Miyajima, M., Watabe, K., Masuda, T., Hagi, K., & Ishigooka, J. (2021). Lurasidone in the long-term treatment of Japanese patients with bipolar I disorder: a 52 week open label study. International journal of bipolar disorders, 9(1), 25.<br><br>Ishigooka, J., Kato, T., Miyajima, M., Watabe, K., Masuda, T., Hagi, K., & Higuchi, T. (2021). Lurasidone in the Long-Term Treatment of Bipolar I Depression: A 28-week Open Label Extension Study. Journal of affective disorders, 281, 160–167.
**Head-to-head study**	Diao, X., Luo, D., Wang, D., Lai, J., Li, Q., Zhang, P., Huang, H., Wu, L., Lu, S., & Hu, S. (2022). Lurasidone versus Quetiapine for Cognitive Impairments in Young Patients with Bipolar Depression: A Randomized, Controlled Study. Pharmaceuticals (Basel, Switzerland), 15(11), 1403.

eTable 4. Sensitivity analysis including major depressive disorder with mixed features

Outcome	Lurasidone dose									
	**10mg**	**20mg**	**30mg**	**40mg**	**50mg**	**60mg**	**70mg**	**80mg**	**90mg**	**100mg**
**Efficacy (SMD)**										
Depression	-0.20 (-0.28,-0.12)*	-0.38 (-0.54,-0.23)*	-0.52 (-0.73,-0.32)*	-0.60 (-0.84,-0.36)*	-0.63 (-0.88,-0.37)*	-0.61 (-0.86,-0.35)*	-0.56 (-0.82,-0.31)*	-0.51 (-0.76,-0.25)*	-0.45 (-0.72,-0.19)*	-0.40 (-0.68,-0.12)*

Abbreviation: SMD: standardized mean difference.

An asterisk with gray background indicates statistical significance.

eTable 5. Number needed to treat from dose-response meta-analysis

Outcome	Lurasidone dose									
	**10mg**	**20mg**	**30mg**	**40mg**	**50mg**	**60mg**	**70mg**	**80mg**	**90mg**	**100mg**
**Efficacy**										
Depression<br>(NNT: decrease)	9<br>(6,18)	5<br>(3,10)	4<br>(3,7)	3<br>(2,6)	3<br>(2,6)	3<br>(2,6)	3<br>(2,7)	4<br>(2,9)	4<br>(3,13)	5<br>(3,26)
Anxiety<br>(NNT: decrease)	16<br>(12,26)	9<br>(6,14)	6<br>(5,10)	6<br>(4,9)	6<br>(4,8)	6<br>(5,9)	7<br>(5,11)	8<br>(5,17)	10<br>(6,52)	14<br>(6,-41)
CGI<br>(NNT: decrease)	8<br>(5,18)	4<br>(3,10)	3<br>(2,7)	3<br>(2,6)	3<br>(2,6)	3<br>(2,6)	3<br>(2,7)	4<br>(2,8)	4<br>(3,11)	8<br>(5,18)
Disability<br>(NNT: decrease)	15<br>(8,59)	8<br>(4,31)	6<br>(3,24)	5<br>(3,22)	5<br>(3,23)	5<br>(3,32)	5<br>(3,74)	6<br>(3,-90)	7<br>(3,-25)	8<br>(3,-13)
Quality of life<br>(NNT: increase)	14<br>(27,10)	8<br>(14,5)	5<br>(9,4)	5<br>(7,3)	4<br>(6,3)	4<br>(6,3)	4<br>(6,3)	4<br>(6,3)	4<br>(7,3)	4<br>(9,3)
**Acceptability**										
Dropout<br>(NNT: decrease)	156<br>(47,-104)	87<br>(27,-54)	69<br>(21,-39)	68<br>(20,-34)	84<br>(22,-32)	141<br>(25,-30)	1526<br>(30,-25)	-147<br>(33,-19)	-68<br>(32,-14)	-44<br>(30,-10)
Mania<br>(NNT: decrease)	-371<br>(308,-100)	-203<br>(167,-47)	-172<br>(122,-34)	-214<br>(99,-32)	-568<br>(83,-37)	487<br>(69,-47)	166<br>(58,-59)	103<br>(49,-67)	78<br>(44,-69)	65<br>(41,-65)
Suicide<br>(NNT: decrease)	464<br>(77,-100)	252<br>(44,-51)	194<br>(35,-38)	182<br>(32,-34)	198<br>(33,-34)	249<br>(36,-35)	394<br>(38,-35)	1203<br>(38,-30)	-1058<br>(35,-23)	-363<br>(31,-18)
Any side effect<br>(NNT: decrease)	-49<br>(-1035,-24)	-25<br>(-306,-12)	-17<br>(-122,-9)	-14<br>(-61,-8)	-12<br>(-37,-7)	-12<br>(-28,-7)	-11<br>(-25,-7)	-11<br>(-29,-6)	-11<br>(-42,-6)	-10<br>(-108,-5)
Akathisia<br>(NNT: decrease)	-123<br>(-1120,-60)	-57<br>(-412,-27)	-36<br>(-188,-17)	-26<br>(-97,-12)	-20<br>(-57,-10)	-16<br>(-38,-9)	-14<br>(-28,-8)	-12<br>(-23,-7)	-10<br>(-20,-6)	-9<br>(-19,-5)
Parkinsonism<br>(NNT: decrease)	11157<br>(121,-102)	-1881<br>(73,-48)	-380<br>(64,-32)	-141<br>(72,-24)	-69<br>(122,-20)	-40<br>(-907,-17)	-26<br>(-84,-13)	-18<br>(-50,-9)	-13<br>(-41,-6)	-10<br>(-37,-4)
**Metabolism / Endocrinology**										
Weight<br>(NNT: decrease)	-22<br>(-49,-14)	-12<br>(-26,-8)	-9<br>(-21,-6)	-9<br>(-22,-6)	-10<br>(-33,-6)	-15<br>(225,-7)	-38<br>(17,-9)	55<br>(8,-11)	16<br>(5,-14)	9<br>(4,-17)
TC<br>(NNT: decrease)	-975<br>(32,-30)	-593<br>(17,-16)	-621<br>(13,-13)	-2059<br>(13,-13)	615<br>(14,-14)	223<br>(16,-18)	128<br>(15,-20)	87<br>(12,-16)	66<br>(8,-11)	53<br>(6,-8)
LDL<br>(NNT: decrease)	215<br>(33,-49)	112<br>(18,-26)	80<br>(14,-21)	66<br>(12,-20)	59<br>(13,-22)	56<br>(13,-26)	55<br>(13,-26)	54<br>(12,-21)	53<br>(10,-15)	52<br>(8,-11)
TG<br>(NNT: decrease)	54<br>(19,-69)	30<br>(11,-37)	25<br>(9,-28)	29<br>(9,-24)	55<br>(12,-21)	-166<br>(18,-15)	-28<br>(22,-9)	-15<br>(21,-5)	-10<br>(17,-4)	-7<br>(14,-3)
Glucose<br>(NNT: decrease)	-700<br>(42,-38)	-241<br>(23,-20)	-106<br>(19,-14)	-54<br>(20,-12)	-32<br>(27,-10)	-22<br>(56,-9)	-16<br>(-5190,-8)	-12<br>(-82,-7)	-10<br>(-57,-6)	-9<br>(-53,-5)
HbA1c<br>(NNT: decrease)	-66<br>(83,-24)	-34<br>(46,-12)	-24<br>(37,-9)	-20<br>(37,-8)	-17<br>(42,-7)	-16<br>(48,-7)	-15<br>(46,-6)	-14<br>(35,-6)	-13<br>(25,-5)	-13<br>(18,-5)
Prolactin, male<br>(NNT: decrease)	-15<br>(-62,-8)	-8<br>(-30,-4)	-5<br>(-19,-3)	-5<br>(-13,-3)	-4<br>(-10,-3)	-4<br>(-8,-3)	-4<br>(-7,-3)	-4<br>(-7,-3)	-4<br>(-7,-3)	-4<br>(-8,-2)
Prolactin, female<br>(NNT: decrease)	-17<br>(-40,-11)	-9<br>(-20,-6)	-6<br>(-14,-4)	-5<br>(-11,-4)	-5<br>(-9,-4)	-5<br>(-8,-4)	-5<br>(-9,-4)	-5<br>(-10,-4)	-6<br>(-14,-4)	-6<br>(-25,-3)

Abbreviation: CGI: clinical global impression; HbA1c: glycohaemoglobin; NNT: number needed to treat; LDL: low-density lipoprotein; TC: total cholesterol; TG: triglyceride.

Positive value indicates number needed to treat for an additional beneficial outcome, while negative value indicates number needed to treat for an additional harmful outcome. Gray background indicates statistical significance.

eTable 6. Detailed quality assessment of included studies using Cochrane risk of bias 2 tool

First Author	Year	Randomization process	Intervention adherence	Missing outcome data	Outcome measurement	Selective reporting	Overall RoB
Loebel	2014a[1]	L	L	L	L	L	L
Loebel	2014b[2]	L	L	L	L	L	L
Suppes	2016[3]	S	L	L	L	L	S
Delbello	2017[4]	L	L	L	L	L	L
Kato	2020[5]	L	L	L	L	L	L

[*] The risk of bias of the additional study with population of major depressive disorder with mixed features was also assessed
H, high risk of bias; L, low risk of bias; RoB, risk of bias; S, some concerns.

eFigure 1. Flowchart of study selection

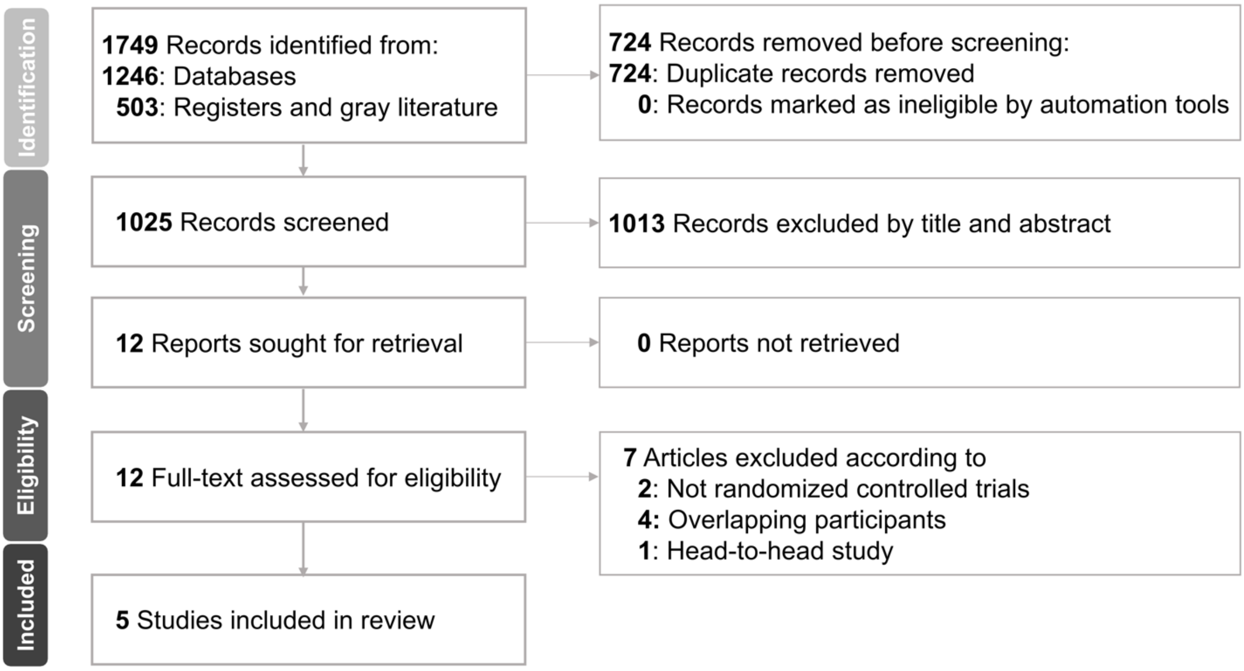

eFigure 2. Summary of quality assessment of included studies using Cochrane risk of bias 2 tool

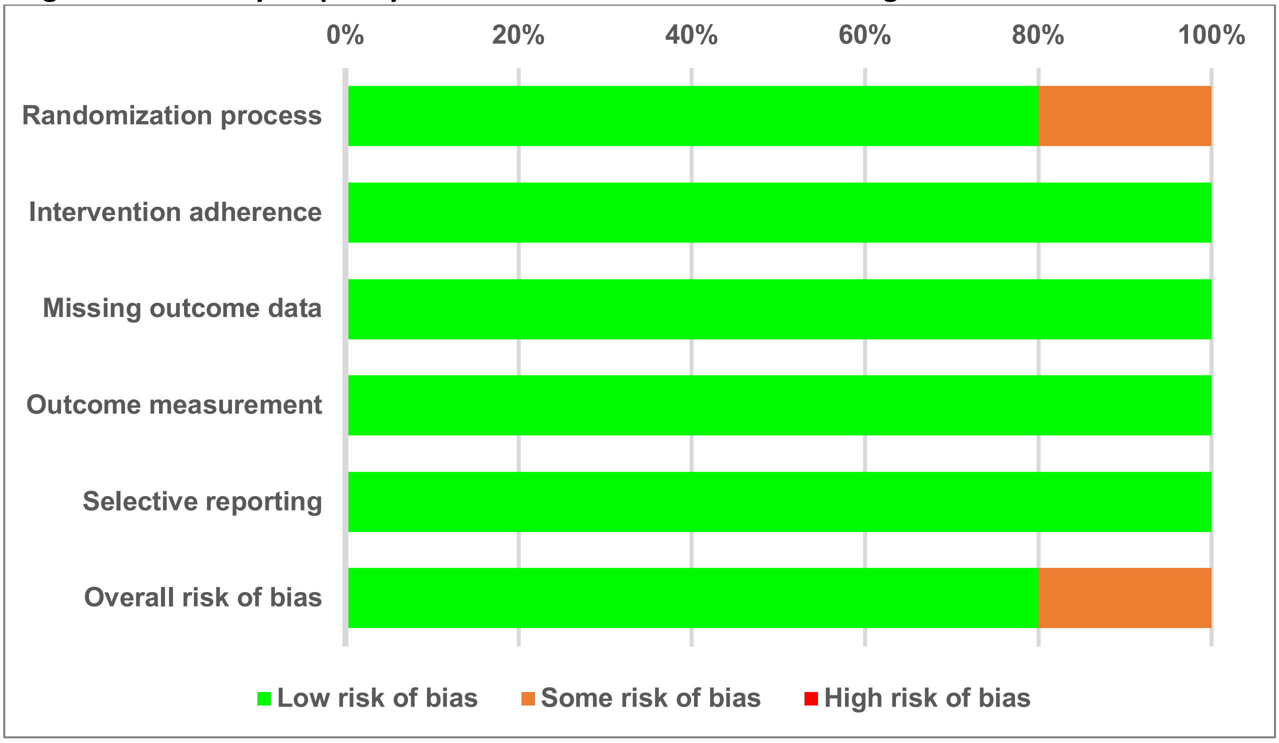

eFigure 3. Variation partition coefficients of dose-response meta-analysis
(A) Depression

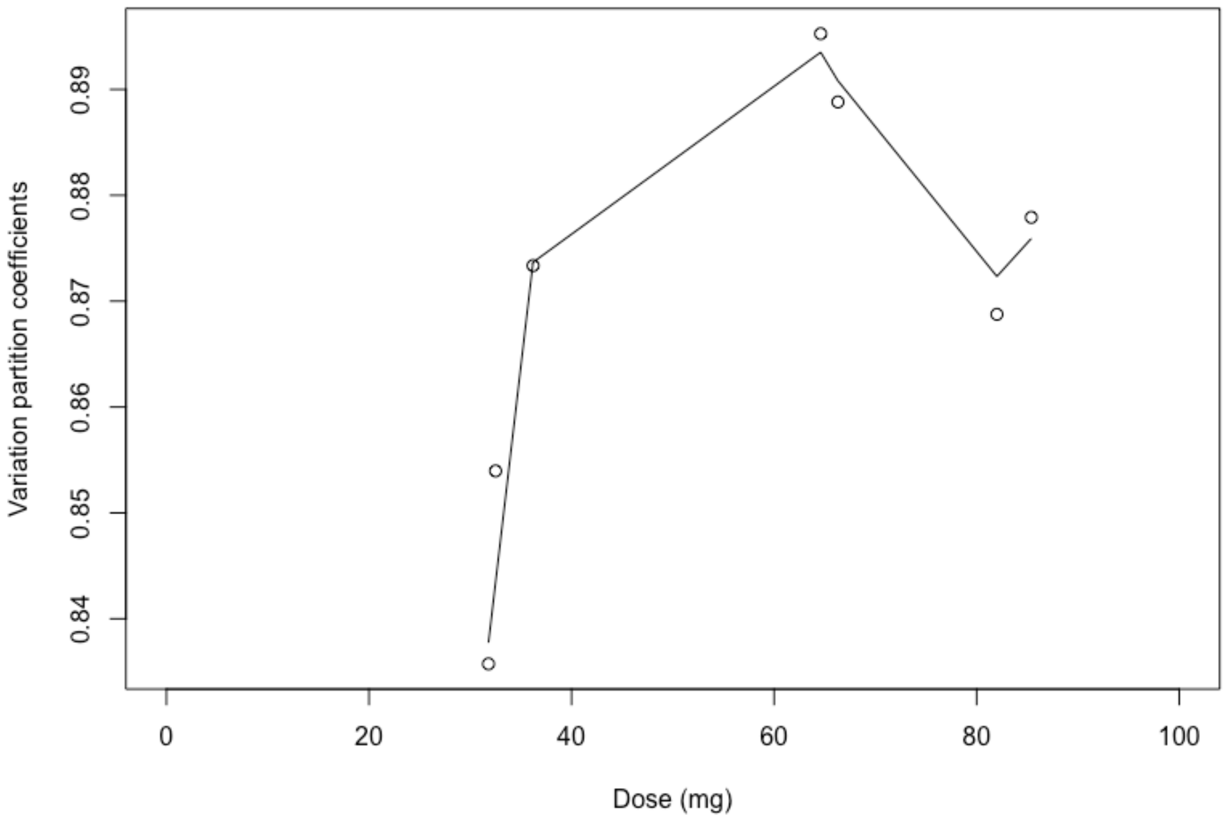

(B) Dropout

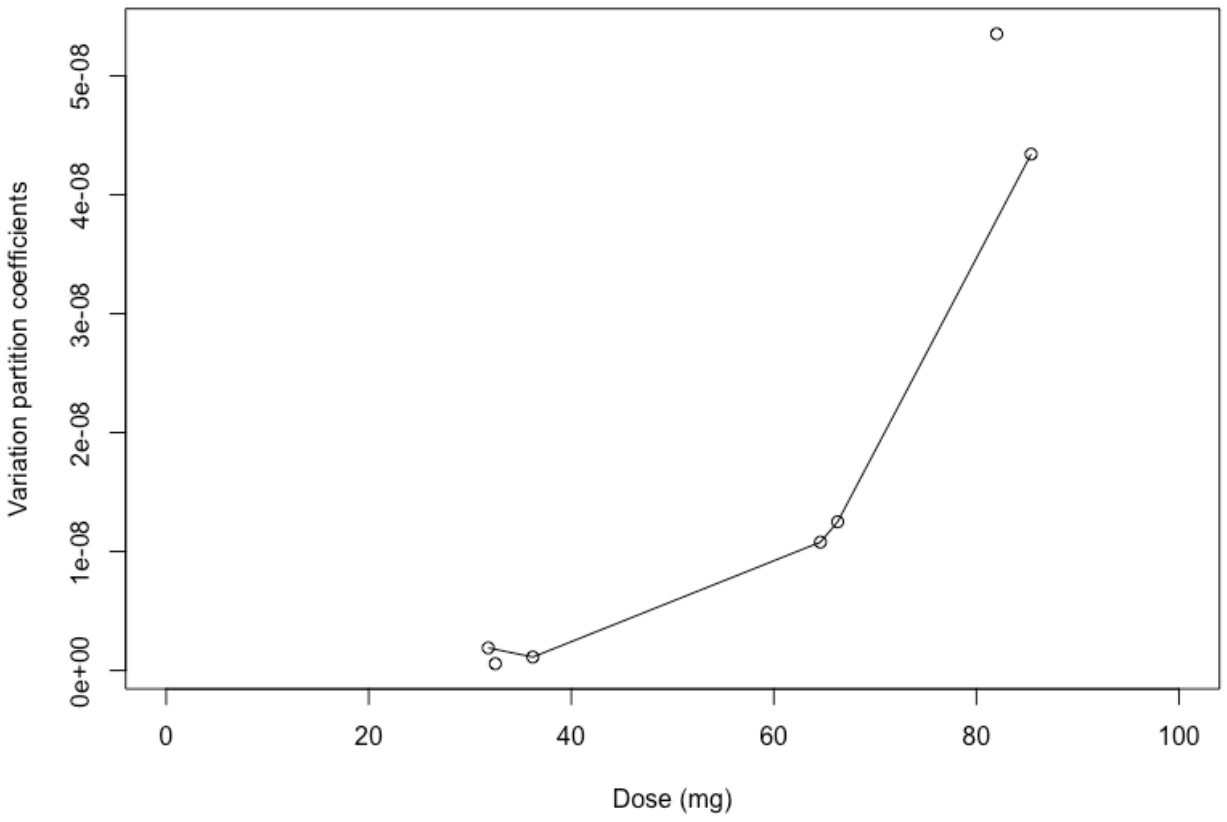

Variation partition coefficients
0e+00
1e-08
2e-08
3e-08
4e-08
5e-08
0
20
40
60
80
100
Dose (mg)

References

1. Loebel A, Cucchiaro J, Silva R, et al. Lurasidone monotherapy in the treatment of bipolar I depression: a randomized, double-blind, placebo-controlled study. Am J Psychiatry 2014a;171(2):160-8. (In eng). DOI: 10.1176/appi.ajp.2013.13070984.
2. Loebel A, Cucchiaro J, Silva R, et al. Lurasidone as adjunctive therapy with lithium or valproate for the treatment of bipolar I depression: a randomized, double-blind, placebo-controlled study. Am J Psychiatry 2014b;171(2):169-77. (In eng). DOI: 10.1176/appi.ajp.2013.13070985.
3. Suppes T, Kroger H, Pikalov A, Loebel A. Lurasidone adjunctive with lithium or valproate for bipolar depression: A placebo-controlled trial utilizing prospective and retrospective enrolment cohorts. J Psychiatr Res 2016a;78:86-93. (In eng). DOI: 10.1016/j.jpsychires.2016.03.012.
4. DelBello MP, Goldman R, Phillips D, Deng L, Cucchiaro J, Loebel A. Efficacy and Safety of Lurasidone in Children and Adolescents With Bipolar I Depression: A Double-Blind, Placebo-Controlled Study. J Am Acad Child Adolesc Psychiatry 2017;56(12):1015-1025. (In eng). DOI: 10.1016/j.jaac.2017.10.006.
5. Kato T, Ishigooka J, Miyajima M, et al. Double-blind, placebo-controlled study of lurasidone monotherapy for the treatment of bipolar I depression. Psychiatry Clin Neurosci 2020;74(12):635-644. (In eng). DOI: 10.1111/pcn.13137.
